# Environmental contamination with taeniid cestode eggs: A systematic literature review

**DOI:** 10.1016/j.fawpar.2025.e00294

**Published:** 2025-10-17

**Authors:** Justine Daudi Maganira

**Affiliations:** Sokoine University of Agriculture, College of Natural and Applied Sciences, Department of Biosciences, P.O. Box 3038, Morogoro, Tanzania

**Keywords:** Taeniid cestodes, *Taenia solium*, Cysticercosis, Vegetables, Soil, Environmental contamination, Diagnostic methods, One health

## Abstract

Taeniid cestodes, including *Taenia solium*, the pork tapeworm, are neglected zoonotic parasites of significant public and veterinary health concern, particularly in low-income countries where sanitation infrastructure is inadequate and pigs and other animals are commonly reared under free-range systems. This review synthesizes published evidence on the occurrence of taeniid eggs or DNA in environmental matrices namely soil, water, vegetables, and sludge, and assesses their role in perpetuating the parasites' transmission cycle. A systematic review of peer-reviewed and indexed literature published between 1989 and 2024 was conducted following PRISMA guidelines. Searches were performed in multiple databases, including PubMed, African Journals Online, and Google Scholar, using keywords related to *Taenia* spp. eggs or DNA in environmental matrices. Articles were included if they reported original research on the detection of taeniid eggs or DNA. Non-English publications, reviews, and studies lacking original data were excluded. Titles and abstracts were screened for relevance, and full texts of eligible articles were reviewed. Data extraction focused on study characteristics, environmental matrices examined, detection methods, and reported prevalence. Contamination levels varied widely by matrix and geography, with higher rates reported in certain parts of Africa, Asia, and Latin America. Vegetables and soil were the most frequently investigated matrices. While contamination in water and sludge remains under-explored; available data suggest they may also play a role in transmission. The findings underscore the critical need for molecular diagnostics to improve species-level identification and inform targeted control strategies. Overall, environmental contamination with taeniid cestode eggs is widespread; however, species-specific evidence for *T. solium* and other *Taenia* spp. remains limited due to the scarce use of molecular diagnostics. These findings highlight the urgent need for molecular studies to accurately identify *Taenia* spp. in environmental matrices. Integrated One Health interventions, including improved sanitation, pig management, public education, and enhanced diagnostic capacity, are essential to reduce taeniid cestodes contamination and mitigate associated zoonotic risks in endemic areas.

## Highlights

• Environmental contamination with taeniid cestode eggs is widespread across soil and vegetables.
• Most studies rely on microscopy, limiting species-specific evidence for Taenia solium.
• Only one study used molecular confirmation (ddPCR) to detect *T. solium* in household soil.
• Water and sludge matrices remain under-studied, though they may play roles in transmission.
• One Health interventions are needed: sanitation, education, diagnostics, and pig management.

## 1 Introduction

Taeniid cestodes (*Taenia* spp.), including *Taenia solium*, are important zoonotic parasites that pose significant veterinary and public health concerns, particularly in low-income countries with inadequate sanitation infrastructure and widespread free-range animal farming (Maganira et al., 2019; Mwang'onde, B. J., Chacha, M. J., and Nkwengulila, G., 2018). The life cycle of taeniid cestodes requires two mammalian hosts. For *T. solium*, humans serve as the definitive host, harboring the adult tapeworm in the intestine, while pigs act as the primary intermediate host, carrying the larval stage (*T. solium* metacestode or cysticercus). Humans can also become intermediate hosts when infected with *T. solium* metacestodes, leading to cysticercosis, including neurocysticercosis, which is a leading cause of acquired epilepsy in endemic regions (Mwang'onde, B. J., Chacha, M. J., and Nkwengulila, G., 2018).

In pigs, cysticercosis results in economic losses in the pig production sector due to the condemnation of infected carcasses at slaughter (Praet et al., 2009). Humans acquire taeniasis, the intestinal infection with adult worms, by consuming raw or under-cooked pork containing viable cysticerci, thereby perpetuating the tapeworm's life cycle. Adult tapeworms produce numerous eggs, which can contaminate the environment through human feces. While pigs may ingest eggs from contaminated feces directly, environmental matrices such as soil, water, vegetables, and sludge primarily pose a risk to humans via indirect transmission (Saelens et al., 2022; Maganira et al., 2020). The widespread presence of taeniid eggs in environmental matrices may facilitate human infection, underscoring the importance of investigating these matrices as potential transmission pathways rather than focusing solely on pig exposure.

Traditional diagnostic methods, such as microscopy, cannot reliably differentiate *T. solium* eggs from other *Taenia* spp. due to morphological similarities. This limitation has resulted in most environmental studies reporting *Taenia* spp. eggs without species-level confirmation, highlighting a critical knowledge gap for species-specific risk assessment (Saelens et al., 2022; Maganira et al., 2020). The presence of taeniid eggs in environmental matrices may play a significant role in sustaining human infection, underscoring the importance of investigating environmental contamination as a component of zoonotic transmission.

Control of taeniid cestode zoonoses, including *T. solium* is challenging due to a combination of socio-economic, cultural, and infrastructural factors, including poor sanitation, open defecation, free-ranging pig husbandry, and inadequate meat inspection practices (Maganira et al., 2019, Maganira et al., 2020; Phiri et al., 2003). These factors facilitate the contamination of environmental matrices, creating indirect pathways for human infection and maintaining transmission in endemic areas. Despite extensive research on cysticercosis in pigs and humans, limited attention has been given to the occurrence and distribution of taeniid eggs in environmental matrices, particularly using molecular tools for species-specific identification.

This review therefore focuses on environmental contamination with taeniid cestode eggs, summarizing current evidence on their occurrence in soil, water, vegetables, and sludge, highlighting methodological limitations, and emphasizing the urgent need for molecular-based studies to specifically detect *Taenia* spp. in environmental matrices. These insights are crucial to inform integrated One Health strategies aimed at reducing zoonotic transmission and associated public health risks.

## 2 Material and methods

A systematic literature review of peer-reviewed and indexed literature published between 1989 and 2024 was conducted following the Preferred Reporting Items for Systematic Reviews and Meta-Analyses (PRISMA) guidelines (Moher et al., 2025). Searches were performed in multiple databases, including PubMed, African Journals Online, and general Google searches, to identify original scientific articles published in English. The search strategy employed Boolean operators and keywords related to the prevalence of taeniid cestode (including *T. solium*) eggs or DNA in environmental matrices. The search syntax was as follows: (“Taeniid cestode” OR “Pork tapeworm” OR “*Taenia solium*”) AND (“eggs” OR “DNA”) AND (“soil” OR “water” OR “vegetable” OR “fruit” OR “sludge”) AND “contamination”. Articles were included if they presented original research data on the prevalence of taeniid cestode eggs or DNA in environmental samples. Non-English articles, studies lacking original data, or those not addressing egg or DNA contamination were excluded. Additionally, references cited within relevant articles were reviewed to identify further studies of interest. All identified articles were screened for duplicates. Titles and abstracts were reviewed to assess relevance. Data extraction and organization were conducted using Microsoft Excel (Microsoft Corp., Redmond, WA, USA), focusing on study characteristics, environmental matrices analyzed, detection methods, and prevalence rates. The extracted data were systematically summarized to identify trends and knowledge gaps related to the environmental contamination of taeniid cestodes. A PRISMA flow diagram illustrating the selection process of studies included in the review was generated using R software version 4.3.3.

## 3 Results

**Fig. 1:**
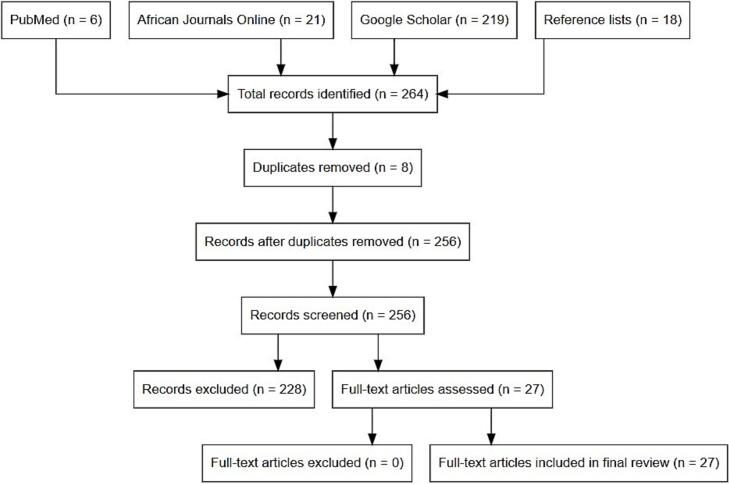
PRISMA flow diagram showing the selection process of studies included in the systematic review of environmental contamination with *taeniid cestode* eggs.

This systematic review identified a total of 264 publications through comprehensive database searches, and screening the references of relevant publications. After applying the exclusion criteria, which included non-English articles and studies lacking original data, 27 studies were deemed eligible and included in the final review. Detailed data extraction and analysis from these studies are presented in Fig. 1.

The reviewed studies provided data on the prevalence, geographical distribution, and detection methods of taeniid cestode eggs or DNA in various environmental matrices, including soil, water, vegetables, and sludge. These studies demonstrated diverse geographical coverage, contamination levels, and detection techniques for taeniid cestodes, with some regions showing particularly high contamination levels in soil and vegetable samples (Table 1, and Table 2).

**Table 1:** Prevalence of taeniid cestodes in environmental matrices.

**Reference**	**Study area**	**Sample size and Matrix**	**Diagnostic method**	**Prevalence**
(Keilbach et al., 1989)	Guerrero State, Mexico	400 soil samples	Sedimentation and microscopy	0 %
(Kozan et al., 2005)	Ankara, Turkey	609 vegetable samples	Washing and microscopy	3.5 %
(Huerta et al., 2008)	Tepetzitzintla, State of Puebla, Mexico	156 soil samples	Flotation and microscopy	24.2 %
(Maikai et al., 2008)	Kaduna, Nigeria	608 soil samples	Flotation and microscopy	36.9 %
(Uga et al., 2009)	Hanoi, Vietnam	317 vegetable samples	Standard washing and microscopy	1.0 %
(Shahnazi and Jafari-Sabet, 2010)	Qazvin Province, Iran	218 vegetable samples	Sedimentation and microscopy	1.8 %
(Hoa et al., 2010)	Hanoi, Vietnam	285 hand-wash samples	Flotation and microscopy	0.7 %
(Al-Megrm, 2010)	Riyadh, Saudi Arabia	470 vegetable samples	Flotation and microscopy	19.7 %
(Abougrain et al., 2010)	Tripoli, Libya	126 vegetable samples	Flotation and microscopy	22.2 %
(Fallah et al., 2012)	Shahrekord, Iran	304 vegetable samples	Washing and microscopy	9.2 %
(Adamu et al., 2012)	Maiduguri, Nigeria	1130 vegetable samples	Washing and microscopy	0.5 %
(Maikai et al., 2012)	Kaduna State, Nigeria	199 vegetable samples	Washing and microscopy	18.3 %
(Adanir and Tasci, 2013)	Burdur, Turkey	111 vegetable samples	Washing and microscopy	2.7 %
(Mwita et al., 2013)	Iringa Rural District, Tanzania	96 soil samples	Flotation and microscopy	6.0 %
(Adenusi et al., 2015)	Ogun State, Nigeria	960 vegetable and fruit samples	Washing and microscopy	1.3 %
(Fallah et al., 2016)	Shahrekord, Iran	901 vegetable samples	Flotation and microscopy	4.7 %
(Asadpour et al., 2016)	Shiraz, southwest Iran	224 vegetable samples	Sedimentation, flotation, staining and microscopy	6.6 %
(Rahmati et al., 2017)	Malayer City, Iran	383 vegetable samples	Flotation and microscopy	1.6 %
(Steinbaum et al., 2017)	Kenya and Bangladesh	200 soil samples	Flotation and microscopy	0 %
(Khan et al., 2017)	Khyber Pakhtunkhwa, Pakistan	520 vegetable samples	Flotation and microscopy	25.0 %
(Amoah et al., 2018)	Durban, South Africa and Dakar, Senegal	60 sludge samples	Sedimentation, flotation and microscopy	Mean egg concentration 54 [±62]
(Maganira et al., 2020) *	Kongwa District, Tanzania	192 Household soil samples	Flotation and microscopy, ddPCR	3.1 %
(Nath et al., 2021)	Sylhet, Dhaka and Chattogram, Bangladesh	240 soil samples	Flotation and microscopy	1.3 %
(Erez et al., 2022)	Afyonkarahisar, Turkey	508 vegetable samples	Sedimentation and microscopy	0.4 %
(Hamza et al., 2022)	Lake Alau, Maiduguri, Nigeria	100 water and 100 soil samples	Sedimentation, flotation and microscopy	0 %
(Shah et al., 2023)	Narowal, Pakistan	145 vegetable samples	Sedimentation, flotation and microscopy	25.5 %
(Sallau et al., 2024)	Kebbi State, Nigeria	50 soil samples	Sedimentation and microscopy	26.0 %

Key: * = Study specifically confirmed *Taenia solium* using molecular methods; all other studies report *Taenia* spp. without species-level confirmation.

**Table 2:** Summary of geographical and environmental contamination with taeniid cestode species.

**Continent**	**Study area**	**Matrix examined**	**Prevalence range**
Africa	Nigeria, Tanzania, South Africa, Libya, Senegal	Soil	0–36.9 %
		Vegetables	0.5–22.2 %
		Water/Sludge	0 – *High egg load* (mean 54 ± 62 eggs/L)
Asia	Iran, Turkey, Pakistan, Vietnam, Bangladesh, Saudi Arabia	Soil	0–1.3 %
		Vegetables	0.4–25.5 %
Latin America	Mexico	Soil	0 %–24.2 %

## 4 Discussion

The findings from the reviewed studies provide valuable insights into the epidemiology of taeniid cestode egg contamination across various environmental matrices, including soil, water, vegetables, and sludge. Several key observations emerge, highlighting both epidemiological trends and methodological limitations that may influence interpretation of contamination levels and zoonotic risks. However, due to methodological constraints, it is difficult to definitively attribute the observed prevalence, for example, to *T. solium*, even in endemic areas. This is because accurate identification requires confirmation through molecular techniques, as the eggs of *T. solium* are morphologically similar to those of other taeniid spp.

### 4.1 Prevalence trends

Only one study (Maganira et al., 2020), which screened village household soil samples using flotation and microscopy, confirmed its findings using molecular techniques (digital droplet PCR (ddPCR)). However, the contamination level of *T. solium* in this study was low (3.1 %). The remaining studies reported the presence of taeniid species eggs without species-level confirmation, making it difficult to attribute the results specifically to any species even in endemic areas.

Reported prevalence rates of taeniid eggs varied widely across environmental matrices and geographical regions (Table 2). In most studies, contamination levels were relatively low (typically <10 %). However, higher rates (22.2 %–36.9 %) were reported in Nigeria (Maikai et al., 2008; Sallau et al., 2024), Mexico (Huerta et al., 2008), Pakistan (Khan et al., 2017; Shah et al., 2023), and Libya (Abougrain et al., 2010). These differences may be attributed to variations in environmental sanitation, free-roaming animals, and the intensity of human and animal infections.

Soil and vegetables were the most frequently studied matrices. Soil contamination showed the greatest variability: while some studies reported high contamination (e.g., >20 % in Nigeria and Mexico), others (Hamza et al., 2022; Keilbach et al., 1989; Steinbaum et al., 2017) found no evidence of taeniid eggs. These inconsistencies likely reflect differences in sampling techniques, soil characteristics, and environmental hygiene.

Vegetable contamination was also prominent. High prevalence rates (18.3 %–25.2 %) were reported in Pakistan (Khan et al., 2017; Shah et al., 2023), Saudi Arabia (Al-Megrm, 2010), and Nigeria (Maikai et al., 2012). In contrast, much lower rates (below 2 %) were observed in Vietnam (Uga et al., 2009), Iran (Rostami et al., 2016), and Nigeria (Adamu et al., 2012). These findings suggest inadequate hygiene practices during cultivation, irrigation with contaminated water, or poor post-harvest handling.

Water and sludge were rarely studied. Only one study (Hamza et al., 2022) investigated water samples and reported a 0 % prevalence. One sludge study (Amoah et al., 2018a) reported a mean egg concentration of 54 (±62) but no prevalence percentage. These results highlight the limited data on these matrices and underscore the need for further research to understand their role in environmental contamination and transmission.

### 4.2 Environmental matrix

Most studies focused on vegetable contamination, likely due to the high risk posed when vegetables are consumed raw or inadequately washed. Prevalence on vegetables ranged from 0.4 % (Erez et al., 2022) to 25.3 % (Shah et al., 2023). Soil contamination ranged from 0 % (Keilbach et al., 1989; Steinbaum et al., 2017) to 36.9 % (Maikai et al., 2008), reflecting significant geographical variation. Few studies addressed water and sludge, but the sludge findings (Amoah et al., 2018b) suggest a potential role in transmission of taeniid eggs through wastewater reuse.

### 4.3 Identification limitations

A major limitation in most reviewed studies is the reliance on flotation and microscopy for identifying *Taenia* spp. eggs. Except for Maganira et al. (2020), which employed ddPCR, all others used conventional microscopy. Due to the morphological similarities among *T. solium* and other taeniid species, accurate species-level identification is not possible without molecular confirmation. Consequently, most studies likely under-estimate or mis-classify *T. solium* prevalence in endemic areas by attributing findings to indistinguishable taeniid species without molecular confirmation. This limitation emphasizes the need for PCR-based techniques to improve diagnostic precision, particularly for *T. solium*, which remains the primary concern in zoonotic transmission.

### 4.4 Geographical distribution

Contamination was predominantly reported in developing countries (Table 2), particularly in Africa (e.g., Nigeria, Tanzania, South Africa, Senegal), Asia (e.g., Pakistan, Iran, Bangladesh, Vietnam), and Latin America (e.g., Mexico). These regions align with the endemicity of taeniid cestodes, especially *T. solium*, often linked to inadequate sanitation, open defecation, and free-roaming pigs. In contrast, lower contamination or none was reported in better-sanitation settings like Turkey (Erez et al., 2022). Addressing contamination in endemic areas requires integrated One Health approaches, including improved sanitation, stricter pig farming regulations, public education, and mass drug administration (MDA).

### 4.5 Sample size variation in studies

Sample sizes ranged from as few as 50 (Sallau et al., 2024) to over 1000 samples (Adamu et al., 2012). Smaller studies are more prone to extreme prevalence estimates due to limited representativeness, while larger studies offer more reliable data. For instance, (Adamu et al., 2012), with 1130 samples, reported a low prevalence of 0.5 %. Future research should include power calculations during study design to determine adequate sample sizes, ensuring accurate and generalizable findings across diverse environments.

### 4.6 Zoonotic transmission and control implications

The detection of taeniid cestode eggs in environmental matrices has significant implications for zoonotic transmission. Contaminated soil and vegetables serve as indirect infection sources, especially in areas with poor sanitation. While not all eggs are *T. solium*, its presence poses serious health risks due to its ability to cause cysticercosis in pigs and humans. This results in economic losses in livestock and serious human health outcomes. Addressing these risks requires improved hygiene, safe food practices, and enhanced wastewater treatment. The continued reliance on microscopy underscores the need for molecular techniques to improve detection and guide interventions. Control measures should include MDA, better pig husbandry, and community health education to reduce taeniasis and cysticercosis. Early detection and surveillance in humans and pigs will help interrupt the transmission cycle and reduce environmental contamination.

### 4.7 Study limitations

This review is limited by the predominance of studies relying on microscopy, potentially mis-classifying taeniid spp.species. Publication bias and exclusion of non-English articles may have also led to under-reporting. Finally, variability in sample sizes and methodologies complicates direct comparison of prevalence across regions.

### 4.8 Conclusions

The reviewed studies provide crucial evidence on the epidemiology of taeniid cestode egg contamination in soil, water, vegetables, and sludge. Findings reveal substantial variability by geography and matrix, with developing countries showing higher contamination levels due to poor sanitation and open defecation. Soil and vegetables were the most frequently contaminated, with some areas reporting notably high prevalence rates. However, the frequent reliance on microscopy without molecular confirmation limits species-specific identification, especially for *T. solium*, the key zoonotic agent. To improve diagnostic accuracy and inform risk assessments, future studies should adopt molecular techniques such as PCR. Integrated One Health strategies such as improved sanitation, food safety, health education, and pig management are essential. Additionally, future research should optimize sample sizes, apply molecular diagnostics, and explore under-studied matrices like water and sludge. These efforts are vital to reducing the burden of taeniid cestodes and associated public and veterinary health risks.

## Funding

This research received no external funding and was self-financed by the author.

## Availability of data and materials

Not applicable.

## Ethics approval and consent to participate

Not applicable.

## Consent for publication

Not applicable.

## CRediT authorship contribution statement

**Justine Daudi Maganira:** Writing – review & editing, Writing – original draft, Methodology, Formal analysis, Data curation, Conceptualization.

## Declaration of competing interest

There is no conflict of interest regarding the publication of this paper.
